# NKp46 defines ovine cells that have characteristics corresponding to NK cells

**DOI:** 10.1186/1297-9716-42-37

**Published:** 2011-02-23

**Authors:** Timothy Connelley, Anne K Storset, Alan Pemberton, Niall MacHugh, Jeremy Brown, Hege Lund, Ivan W Morrison

**Affiliations:** 1 The Roslin Institute, Royal (Dick) School of Veterinary Studies, University of Edinburgh, Easter Bush, Midlothian, Edinburgh, Scotland, EH25 9RG, UK; 2 Department of Food Safety and Infection Biology, Norwegian School of Veterinary Science, P.O. Box 8146, Dep, N-0033 Oslo, Norway; 3 Reproductive Biology, The Queens Medical Research Institute, University of Edinburgh, Edinburgh, Scotland, EH16 4TJ, UK

## Abstract

Natural killer (NK) cells are well recognized as playing a key role in innate immune defence through cytokine production and cytotoxic activity; additionally recent studies have identified several novel NK cell functions. The ability to study NK cells in the sheep has been restricted due to a lack of specific reagents. We report the generation of a monoclonal antibody specific for ovine NKp46, a receptor which in a number of mammals is expressed exclusively in NK cells. Ovine NKp46^+ ^cells represent a population that is distinct from CD4^+ ^and γδ^+ ^T-cells, B-cells and cells of the monocytic lineage. The NKp46^+ ^cells are heterogenous with respect to expression of CD2 and CD8 and most, but not all, express CD16 - characteristics consistent with NK cell populations in other species. We demonstrate that in addition to populations in peripheral blood and secondary lymphoid organs, ovine NKp46^+ ^populations are also situated at the mucosal surfaces of the lung, gastro-intestinal tract and non-gravid uterus. Furthermore, we show that purified ovine NKp46^+ ^populations cultured in IL-2 and IL-15 have cytotoxic activity that could be enhanced by ligation of NKp46 in re-directed lysis assays. Therefore we conclude that ovine NKp46^+ ^cells represent a population that by phenotype, tissue distribution and function correspond to NK cells and that NKp46 is an activating receptor in sheep as in other species.

## Introduction

Natural killer (NK) cells are lymphocytes of the innate immune system which through production of cytokines and cytotoxic activity are capable of offering an immediate response to pathogen-infected and transformed host cells [1]. NK cells recognise potential targets through a diverse repertoire of germ-line encoded activating and inhibitory receptors including members of the killer cell Ig-like receptor (KIR), Ly49, and CD94:NKG2 families and the natural cytotoxicity receptors (NCRs) NKp46, NKp30 and NKp44. Induction of NK cell function is dependent on the relative balance of signals received from activating and inhibitory receptors engaged upon interaction with target cells. Through interactions with other cells of the immune system, NK cells have also been found to regulate the development of both innate and adaptive immune responses in a variety of ways. This includes the activation/maturation of antigen-presenting cells [2], providing IFNγ for the priming of T_H_1 CD4^+ ^T-cells [3], modulating the function of T_reg _cells [4] and exerting an immunoregulatory effect via the production of IL-10 [5].

Studies in a range of mammalian species have confirmed that NKp46 expression is restricted to NK cells and that it serves as the most reliable NK cell marker available [6-9]. NKp46 is a type I transmembrane glycoprotein with 2 extracellular C2-type Ig-like domains that associate via an arginine residue in the transmembrane region with the ITAM bearing molecules CD3ζ and FcεRIγ [6,9,10]. In humans, NKp46 has been shown to be a principal activating receptor against a variety of NK cell targets [10,11]. However, with the exceptions of the haemagglutinin of influenza virus and the haemagglutinin-neuraminidase of parainfluenza virus and Newcastle disease virus the ligands for NKp46 are currently unknown [12,13].

The generation of a bovine NKp46-specific antibody has facilitated the study of NK cells in cattle and shown that they contribute to the response against a variety of pathogens including *Mycobacterium bovis, Babesia bovis *and *Neospora caninum *[14-16]. At present there is no equivalent antibody in sheep and most previous work has been restricted to the description of NK-like cytotoxicity in ovine PBMC and endometrial cell populations [17-20], although a recent publication has demonstrated that circulating CD16^+^/CD14^- ^cells in ovine PBMC have the morphological and functional characteristics of NK cells [21]. In this paper we describe the generation of a monoclonal antibody specific for ovine NKp46 and show that cells expressing NKp46 have a phenotype, tissue distribution and cytotoxic function characteristic of NK cells.

## Materials and methods

### Animals and tissue preparations

Samples were taken from sheep of various breeds aged between 3 months and 1 year. PBMC were isolated from blood collected in EDTA by density gradient centrifugation (900 × *g*, 30 min) over Ficoll-Paque Plus (Amersham Biosciences, Little Chalfont, UK) and washed three times in PBS/2 mM EDTA. Single-cell suspensions from spleens and lymph nodes were obtained by passing the products of dilacerated tissues through a mesh with a 50 μM pore size (BD, San Jose, CA, USA). Tissue samples for immunohistochemical analysis were snap frozen in isopentane/dry ice and mounted in optimal cutting temperature (OCT) compound (Tissue-Tek, Sakura-Finitek, Zoeterwoude, The Netherlands). For samples from the lung, isolated lobes were inflated with a mixture composed of 30% sucrose in water (w/v) and OCT at a ratio of 2:1 prior to snap freezing. Tissue sections cut to 7 μm thickness were mounted on poly-L-lysine coated slides and stored at -80°C until use.

### Cloning of the ovine NKp46 gene

The NCBI *Ovis aries *trace archives [22] were searched in May 2007 for sequences orthologous to bovine/human NKp46 (GenBank AF422181/NM_004829) using the BLASTn algorithm.

Total RNA was extracted from ovine PBMC using Tri-reagent (Sigma-Aldrich, Poole, Dorset, UK) and cDNA subsequently synthesized using the Reverse Transcription System (Promega, Madison, WI, USA) with priming by the Oligo (dT)_15 _primer, according to the manufacturer's instructions. Based on sequence data obtained from the NCBI bovine WGS archive database primers in the 5' (tcactcaccacatcctgagc) and 3' (cttcctccatgggttccac) UTR of the bovine NKp46 gene were designed and used in PCR reactions composed of 2.5 μL cDNA, 25 pmol of each primer, 2.5 units of Biotaq (Bioline, London, UK), 5 μL SM-0005 10× buffer (ABgene, Epsom, Surrey, UK) and water to give a final volume of 50 μL. The cycling conditions for these reactions were 3 min at 95°C, 35 cycles of 1 min at 94°C, 1 min at 55°C, 1 min at 72°C and a final extension period of 10 min at 72°C. PCR products were purified and sub-cloned using the pGEM-T Easy vector system (Promega, Madison, WI, USA) according to the manufacturer's instructions and representatives sequenced (DBS Genomics, University of Durham, UK).

DNA and predicted protein sequence analysis was performed using DNAsis Max vr2.7 software (Miraibio, Alameda, CA, USA) and the tools accessed through the InterProScan [23] and Scratch Protein Predictor [24] websites.

### Generation of an anti-ovine NKp46 mAb

The extracellular region of the ovNKp46 gene was amplified from ovine PBMC by PCR using 5' (aagcttttaccaggcagaatctgag) and 3' (ggatccccagaggaaatggtcttt) primers that incorporate HindIII and BamHI restriction sites respectively in reactions composed of 2.5 μL cDNA, 25 pmol of each primer, 2.5 units of Biotaq, 5 μL SM-0005 10× buffer and water to give a final volume of 50 μL and run under the following conditions: 3 min at 95°C, 32 cycles of 1 min at 94°C, 1 min at 58°C, 1 min at 72°C and a final extension period of 5 min at 72°C. The product was purified, cloned into pGEM-T Easy, released by HindIII/BamHI digestion and then inserted into a mammalian expression vector containing the hinge, CH2 and CH3 regions of the murine IgG2b gene (kindly provided by HC Aasheim, The Norwegian Radium Hospital, Oslo, Norway) as previously described [7,25]. Briefly, 40 μg of construct was mixed with 160 μL of Lipofectamine LTX (Invitrogen, Paisley, UK) and used to transiently transfect 293T cells at ~80% confluence in 162 cm^2 ^flasks, cells were then grown in serum-free AIM-V media (Invitrogen) for four days. The secreted ovNKp46-mFcγ2b fusion protein was then purified on a protein G column (Amersham Biosciences) according to the manufacturer's instructions, quantified using a BCA assay (Thermo Scientific, Rockford, IL, USA) and assayed by both sodium dodecyl sulphate-polyacrylamide gel electrophoresis (SDS-PAGE) and MALDI-TOF (data not shown).

Young, female BALB/c mice were immunized with three injections: primary and secondary subcutaneous injections of 100 μg and 25 μg protein respectively in TitreMax Gold (TitreMax Inc, Norcross, GA, USA), and a final intra-peritoneal injection of 25 μg protein in PBS. Spleen cells were fused with X63 cells by conventional techniques and hybridoma clones screened by flow cytometry against P815 cells that had been permanently transfected with a pFLAG-CMV-3 expression vector (Sigma-Aldrich, Dorset, UK) containing the complete coding region of ovNKp46 gene. One hybridoma clone was sub-cloned by limiting dilution and the resulting hybridoma named EC1.1.

### Antibodies, flow cytometry, and fluorescent immunohistochemical analysis

All primary monoclonal antibodies used in this study were murine, with the exception of SW73.2 which was produced in a rat: 36F (ovine CD2:IgG2a [26]), SW73.2 (ovine MHCII: rat IgG2b [27]), both obtained from the Moredun Research Institute, UK; 44.38 (ovine CD4: IgG2a [28]), CC126 (bovine CD11b: IgG2b [29]), both obtained from AbD Serotec, Oxford, UK; δG9 (human perforin: IgG2b) and 36/E-cadherin (human E-cadherin: IgG2a) both from BD Biosciences (Erenbodegen, Belgium); VPM13 (ovine IgM: IgG2b - a gift from Bernadette Dutia, Centre for Infectious Diseases, University of Edinburgh), KD1 (human CD16: IgG2a - a gift from Daniela Pende, Istituto Nazionale per la Ricerca sul Cancro, Genova, Italy), CC63 (bovine CD8: IgG2a [30], CC15 (bovine WC1: IgG2a [31]) and ILA-111 (bovine CD25: IgG1 [32]). Alexa Fluor 488-conjugated murine IgG1-specific, PE-conjugated murine IgG2a-, murine IgG2b- and rat IgG- and Alexa Fluor 647-conjugated murine IgG2a-specific secondary antibodies were all obtained from Molecular Probes (Invitrogen). Isotype controls (all from AbD Serotec) were included in each experiment.

Two-colour and single colour flow cytometric analysis was performed using a FACScalibur flow cytometer with Cellquest software (BD, Franklin Lakes, USA), gated to include viable cells according to forward and side scatter parameters. For detection of intra-cellular perforin, cells were permeabilised using the Cytofix/Cytoperm kit according to the manufacturer's instructions (BD Bioscience). Optimal concentrations of all primary and secondary antibodies were determined in preliminary titration assays.

For multi-colour immunofluoresence sections were air dried prior to fixing in methanol for 10 min at -20°C. Sections were then washed twice in PBS/0.5% Tween 80 and blocked by incubation in 10% normal goat serum in PBS/0.5% Tween 80 for 30 min at room temperature. Slides were then mounted into Sequenza immunostaining units and incubated with primary antibodies at 4°C overnight. Following two washes in PBS/0.5% Tween 80 slides were incubated with fluorophore conjugated secondary antibodies for 1 h at room temperature. After a final wash, slides were mounted in Mowiol mounting medium (Calbiochem-Novabiochem; San Diego, CA, USA), and images taken using a SPOT RT3 camera (Diagnostic Instruments, Sterling Heights, USA) mounted on an Axiovert 100 inverted microscope (Carl Zeiss; Welwyn Garden City, UK) and analysed using SPOT software (Diagnostic Instruments). All antibodies were diluted in 10% normal goat serum in PBS/0.5% Tween 80 at the optimal concentrations as determined in preliminary titration assays.

### Cloning and expression of recombinant ovine IL-2

mRNA was extracted from ConA stimulated ovine PBMC (μMACS kit, Miltenyi Biotec, Bergisch Gladbach, Germany), followed by complementary DNA synthesis by SuperScriptIII reverse transcriptase and Oligo(dT)_20 _primer (both Invitrogen). As information on the sequence in the 5' UTR of the ovine IL-2 was not available, a forward primer designed to recognize 15 nucleotides upstream of the bovine IL-2 start codon to include the translational initiation region and that also included the BamHI restriction site was used; 5'-ggatcctcaactcctgccacaatgta-3'. The reverse primer was 5'-ctcgagttaagactaacagttacaaaaggt-3' including XhoI in the 5' end and recognizing the sequence 89 nucleotides into the 3'UTR of ovine IL-2. (GenBank NM_001009806.1). The ovine IL-2 sequence was amplified by PCR using PfuUltra™(Stratagene, La Jolla, CA, USA) at 3 min at 94°C, 35 cycles of 45 s at 94°C, 30 s at 60°C, 1.5 min at 72°C and a final extension period of 10 min at 72°C. The obtained PCR product of 608bp was purified, cloned into 2.1-TOPO vector, released by BamHI/XhoI digestion and then inserted into pcDNA1 vector (Invitrogen). The correctness of the construct was verified by sequencing. Plasmid DNA was transfected into 293T cells, ovine IL-2 was harvested in RPMI medium containing 10% FCS and the activity was determined in a proliferation assay of ConA stimulated ovine PBMC as described earlier for bovine IL-2 [33]. The supernatant containing 1 × 10^4^U rOvIL-2/mL was stored at -20°C and used without further purification at the indicated concentration.

### Isolation and culture of NKp46^+ ^cells

PBMC re-suspended at 5 × 10^7 ^cells/mL in PBS/0.5%BSA/2 mM EDTA were incubated with EC1.1 supernatant (final dilution of 1:4, based on preliminary titration analysis) for 30 min at 4°C. After two washes the cells were incubated with DynaBead pan Mouse IgG (Invitrogen) at 4 × 10^6 ^beads/mL for 30 min at 4°C with shaking. Antibody-labelled cells were magnetically isolated using a DynaMag-15 (Invitrogen) and washed three times. Isolated cells were cultured in RPMI1640 supplemented with 100 U/mL penicillin, 100 μg/mL streptomycin, 292 μg/mL L-glutamine, 1 mM sodium pyruvate, 50 μM 2-mercaptoethanol, 10% foetal calf serum (all from Invitrogen), 20 ng/mL recombinant human (rHu) IL-15 (R&D Systems, Minneapolis, USA) and rOvIL-2 at 200 U/mL. Cultures were incubated at 37°C in 5% CO_2 _for 7-8 days, with the magnetic beads removed after 48 h and media/cytokine supplemented every 2-3 days as required. Flow cytometric analysis on day 7-8 demonstrated that all cultures were > 93% NKp46^+^.

### Cytotoxicity assays

Cytotoxic activity of cultured ovine NKp46^+ ^cells was examined using 4 h ^111^In-release assays. In brief, YAC-1, K562 and P815 target cells were re-suspended at 1 × 10^7^/mL and 50 μL aliquots were incubated with ^111^Indium oxine (Amersham Biosciences) at 37°C for 30 min. After six washes in RPMI 1640/5% foetal calf serum, aliquots of 2.5 × 10^3 ^labelled target cells were placed into 96-well V-bottomed plates. In duplicate, two fold dilutions from 2 × 10^4 ^to 1.25 × 10^3 ^of NKp46^+ ^effector cells (day 7-8 of culture) were added to the labelled target cells giving effector to target cell ratios of 8:1 to 0.5:1. After incubation at 37°C for 4 h supernatant was harvested and ^111^In-release measured in a Wallac Wizard 1470 Automatic Gamma Counter (PerkinElmer, Bucks, UK). Percentage specific lysis was calculated as ((sample release-spontaneous release) × 100%/(maximal release-spontaneous release)) and expressed as the mean of the duplicated assays. Maximal and spontaneous release were derived from triplicates of target cells incubated in 0.2% Tween20 and RPMI1640/5% foetal calf serum, respectively. In re-directed lysis assays P815 cells were incubated in EC1.1 supernatant or an isotype matched antibody for 30 min at room temperature before the final (i.e. sixth) wash removing the ^111^Indium oxine label.

## Results

### The ovine NKp46 gene

**Figure 1:**
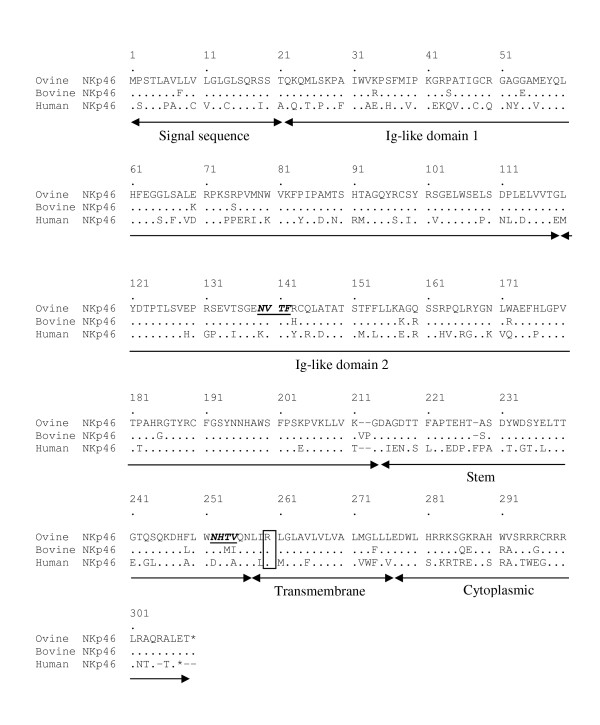
**Alignment of the amino acid sequences of ovine, bovine and human NKp46**. Identity of residues between sequences is indicated with a dot and gaps by a dash. The location of the signal sequence, two extracellular Ig-like domains, stalk, transmembrane region and intra-cytoplasmic region have been annotated. The sites of potential N-linked glycosylation are in bold, underlined script and the conserved arginine residue in the transmembrane region is boxed.

Examination of ovine sequence databases by probing with the bovine and human NKp46 nucleotide sequences only identified sequences orthologous to human NKp46 exons 4 and 5 (data not shown). To obtain complete sequence of the ovine NKp46 coding region, cDNA from ovine PBMC was amplified using PCR primers specific to the 5' and 3' UTR of the bovine NKp46 gene and the products cloned. The consensus sequence (GenBank accession number HQ433587) of these clones predicts a polypeptide product that shows 92.5% and 60.9% identity with bovine and human NKp46 respectively (Figure 1). Following removal of the predicted signal sequence (1-21aa), the mature polypeptide of 285 amino acids contains two extra-cellular Ig-like domains (25-199aa and 120-212aa), an extra-cellular stem (213-254aa), a transmembrane region (255-273aa, which includes a conserved arginine) and a cytoplasmic tail (274-285aa). The predicted molecular mass of the mature unglycosylated protein is 32.2kDa, similar to that of the bovine and human orthologues [7,10]. However, predicted N-glycosylation sites at Thr139 and Thr249 suggests that the molecular mass of the native protein is likely to be higher.

### Generation of an ovine NKp46-specific monoclonal antibody

**Figure 2:**
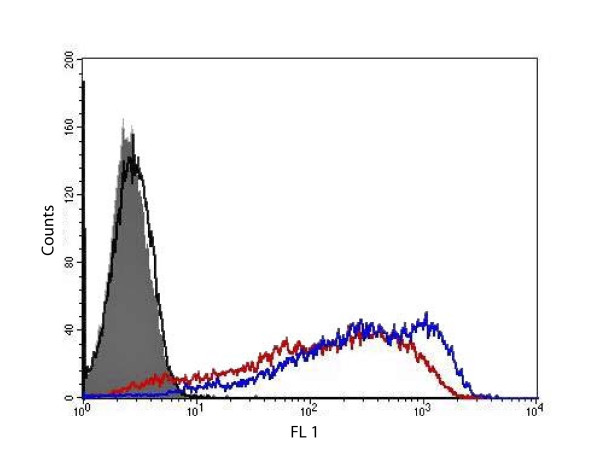
**EC1.1 is specific for ovine NKp46**. P815 cells transfected with an ovNKp46-FLAG construct are stained similarly by supernatant from EC1.1 (blue line) and a FLAG-specific antibody (red line) but not by an isotype control antibody (black line). Untransfected P815 cells are not stained by EC1.1 supernatant (solid grey).

Mice were immunised with a fusion protein composed of the extra-cellular region of ovine NKp46 and the hinge and Fc region of murine IgG2b. Screening of hybridomas derived from these mice for reactivity against P815 cells transfected with an ovine NKp46-FLAG construct identified several positive hybridomas. A cloned derivative of one of these hybridomas, termed EC1.1, was obtained and found to produce an IgG1 isotype antibody that specifically recognised ovine NKp46 (Figure 2).

### The phenotype of ovine NKp46^+ ^cells

**Figure 3:**
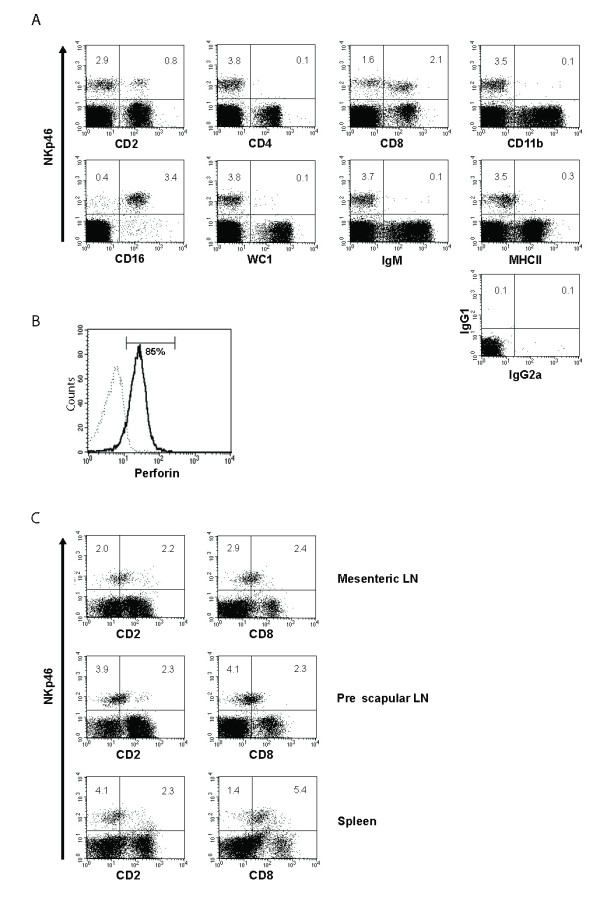
**Analysis of the phenotype of ovine NKp46^+ ^populations**. (A)Two-colour flow cytometry analysis of ovine PBMC co-stained with EC1.1 and a selection of monoclonal antibodies against CD2, CD4, CD8, CD11b, CD16, WC1, IgM and MHCII. WC1 and IgM are surface molecules expressed by subsets of γδ T-cells and B-cells respectively. (B) Perforin expression by NKp46^+ ^PBMC. Cells expressing NKp46 were stained with anti-perforin monoclonal antibody (solid line) or an isotype control (broken line). (C) Two-colour flow cytometry analysis of mesenteric and pre-scapular lymph nodes and the spleen cell populations co-stained with EC1.1 and monoclonal antibodies against CD2 and CD8. The results shown are representative of the results obtained from a minimum of five animals.

Flow cytometry analysis of samples from six animals showed that NKp46 was expressed on between 3-16% of PBMC. Using 2-colour flow cytometry these cells were found to have a CD4^-^, WC1^-^, IgM^-^, MHCII^- ^phenotype indicating that NKp46 is expressed on a cell subset that is distinct from CD4 T-cells, γδ T-cells, B cells and cells of the monocytic lineage (Figure 3a). CD2 expression defined two distinct NKp46^+ ^subsets - a minor (15-26%) CD2^hi ^subset and a larger (74-85%) CD2^lo/- ^population. Similarly, CD8 expression defined a CD8^lo/- ^subset (40-55%) and a CD8^hi ^subset (45-60%). The majority (75-88%) of the NKp46^+ ^cells were positively stained by the monoclonal antibody KD1 which is specific for human CD16 and has been found to cross-react with the bovine orthologue of this gene and identifies populations within ovine PBMC [21,34]. Intra-cellular staining identified the presence of perforin, a protein present in cytotoxic granules of NK cells and reflecting their cytotoxic potential, in the majority of NKp46^+ ^cells (Figure 3b). In contrast to human and murine NK cells, the ovine NKp46^+ ^population did not express CD11b (Figure 3a).

### Isolation and in vitro culture of ovine NKp46^+ ^cells

**Figure 4:**
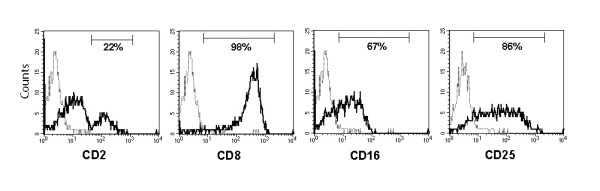
**Phenotype of cultured NKp46^+ ^populations**. Ovine NKp46^+ ^populations after 7-8 days of in vitro culture were stained with monoclonal antibodies against CD2, CD8, CD16 and CD25 (solid lines) or isotype controls (broken lines). Results are shown from one culture that was representative of those derived from five animals.

Using EC1.1 and immunomagnetic separation NKp46^+ ^cells from PBMC were isolated and then cultured in vitro in media containing rOvIL-2 and rHuIL-15 to derive purified NKp46^+ ^populations. Phenotypic analysis revealed that the cultured NKp46^+ ^cells were uniformly CD8^+ ^and CD16^lo/-^, whilst expression of CD2 defined two distinct subsets - a major CD2^lo/- ^population (77-85%) and a minor CD2^hi ^population (15-23%) (Figure 4). CD25 was expressed on > 60% of the cells indicating that the majority of the cells in the cultured populations were activated.

### In vitro cultured ovine NKp46^+ ^cells exhibit cytotoxic activity

**Figure 5:**
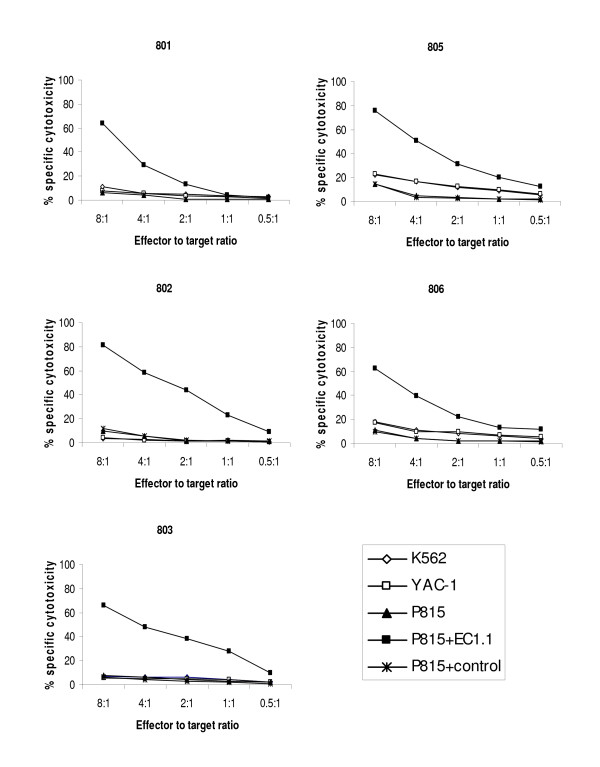
**Cultured Ovine NKp46^+ ^cells exhibited cytotoxic activity**. Cytotoxicity of NKp46^+ ^cultures from five animals as determined in 4 h-^111^In release assays. Lysis of the xenogenic NK-sensitive cells lines K562 and YAC1 exhibited inter-animal variation, with lysis ranging from approximately 3-22% at effector to target ratios of 8:1. Direct lysis of P815 cells (FcγR^+^) was low (6-14% at effector to target ratios of 8:1), but in redirected cytotoxicity assays pre-incubation with anti-NKp46 antibody (EC1.1) dramatically increased cytotoxic activity (62-81% at effector to target ratios of 8:1) of all NKp46^+ ^cultures. Pre-incubation with an isotype control antibody did not elevate cytotoxicity above that seen in the absence of antibody.

The cytotoxic activity of NKp46^+ ^cultures from five animals were examined in standard 4 h In^111^-release assays (Figure 5). The levels of cytotoxicity against YAC-1 and K562 were generally low but exhibit some inter-animal variation, with cytotoxicity at an effector to target ratio of 8:1 ranging from ~3-22% between individuals. Lysis of murine FcγR-expressing P815 cells was also low (ranging from 6-14% at an effector to target ratio of 8:1). However, for all five cultures pre-incubation of the P815 target cells with the EC1.1 antibody induced greatly enhanced levels of cytotoxic activity (ranging from 62-81% at an effector to target ratio of 8:1). This increase in lysis of P815 was not seen when pre-incubated with an isotype controlled antibody. These re-directed lysis assay results demonstrate that that all of the NKp46^+ ^cultures had cytotoxic capability and that the NKp46 receptor functions as a cytotoxicity activating receptor in ovine NK cells.

### Ovine NKp46^+ ^cell distribution in lymph nodes and mucosal surfaces

In other species NK cells have a wide distribution, particularly in secondary lymphoid tissue and sites of potential pathogen entry. Using EC1.1 we examined the distribution of ovine NKp46^+ ^cells in mucosal and non-mucosal lymph nodes and in the lungs, gastro-intestinal tract and non-gravid uterus of sheep.

**Figure 6:**
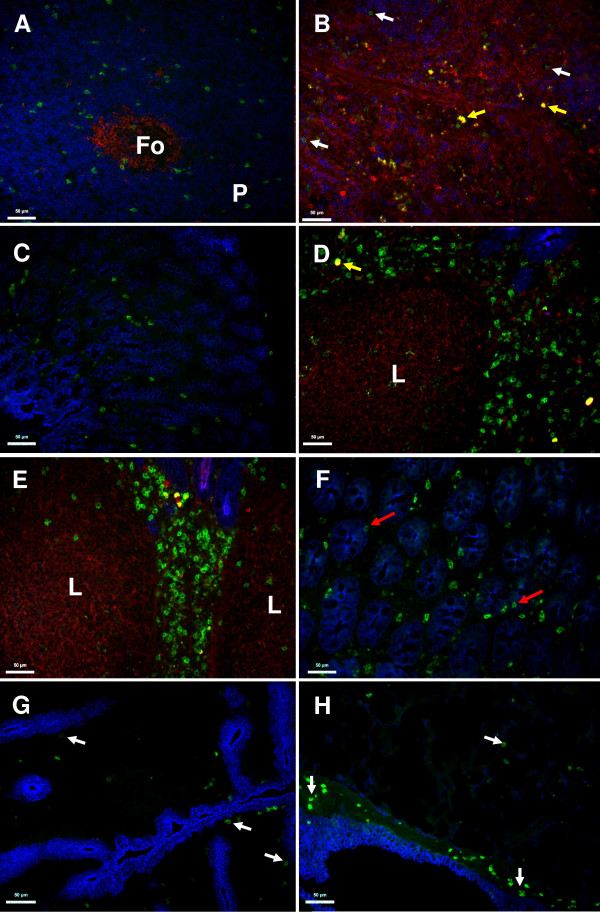
**Distribution of ovine NKp46^+ ^cells in lymph nodes and mucosal surfaces**. Sections from ovine pre-scapular lymph node (A - cortex and B - medullary zone), abomasum (C), duodenum (D), ileum (E), colon (F), uterus (G) and lung (H). NKp46^+ ^cells: green; B-cells (IgM^+^) red; CD4^+ ^T-cells blue in panel A and B; Epithelial cells (E-cadherin^+^) blue in parts C-H. White arrows: individual NKp46^+ ^cells. Red arrows: putative intra-epithelial NKp46^+ ^cells. "Fo": follicle and "P": paracortex. "L": lymphocytic aggregate. Isotype matched controls demonstrated diffuse and dull background fluorescence in both the red and green channels (data not shown), however this could easily be distinguished by character and/or intensity from the bright cell-surface staining seen with the NKp46 and IgM-specific antibodies. Autofluorescent artifacts such as those demonstrated by the yellow arrows (e.g. in C and D) were not uncommon in the tissues examined. A scale for each of the figure parts is given. Images are taken from tissues that were representative of samples taken from a minimum of three animals as determined by preliminary single colour immuncytochemistry analysis.

Immunohistochemical analysis demonstrated that the distribution of NKp46^+ ^cells in mucosal and non-mucosal lymph nodes was similar; NKp46^+ ^cells were located in both the para-cortical and medullary areas but only rarely identified within the follicles (Figure 6a and 6b). Examination of the gastro-intestinal tract (GIT) revealed that NKp46^+ ^cells were absent from the rumen (data not shown) but were present sporadically throughout the lamina propria of the abomasum, duodenum, jejunum, ileum and colon, with occasional NKp46^+ ^cells having an intra-epithelial location (e.g. Figure 6c and 6f). Additionally it was notable that NKp46^+ ^cells were scarce in B-cell aggregates or follicles present in the various compartments of the gut but were present in high densities in the inter-follicular areas (e.g. Figure 6d and 6e). NKp46^+ ^cells were also evident in sub-epithelial locations in the uterus (Figure 6g) and in the lung were found in the sub-epithelium of airways and also within the interstitium (Figure 6h).

NKp46^+ ^cells in the pre-scapular and mesenteric lymph nodes constituted 2.6-6.2% and 2.2-4.2% of the mononuclear cell population respectively, and were also found at a similar frequency in the spleen (3.2-4.9%). The phenotype of these populations were generally consistent with that of the NKp46^+ ^cells in PBMC but tended to demonstrate a more homogenous CD2^lo ^and CD8^lo ^expression (Figure 3c).

## Discussion

Research into ovine NK cells has been impeded by a lack of specific reagents. Indeed, until recent work by Elhmouzi-Younes et al. [21], identifying the CD16^+^/CD14^- ^subset of ovine PBMC as NK cells, studies in sheep had been confined to the description of NK-like function in unfractionated lymphocyte populations from blood and uterine tissue [17-20]. In this study we report the generation of a monoclonal antibody specific for ovine NKp46, a cell surface marker specifically expressed by NK cells in a variety of species including humans, primates, mice, rat and cattle and that has been proposed as the most definitive marker for NK cells in mammals [6-9,35]. We have demonstrated that the phenotype, distribution and function of the ovine NKp46^+ ^cells identified with this antibody correspond with those previously described for NK cells in other species.

NK cells form a phenotypically heterogenous population, with subsets of distinct phenotypes frequently associated with particular functions and/or anatomical location [36]. Comprehensive inter-species comparisons of NK cell phenotype is precluded by differences in NK cell markers expressed in individual species [35]. However the phenotype of ovine NKp46^+ ^cells in PBMC and secondary lymphoid tissues (spleen, mucosal and non-mucosal lymph nodes) was consistent with that of NK cells in other species in several respects. Firstly, NKp46^+ ^cells formed subsets that were distinct from CD4^+ ^T-cell, γδ^+^T-cell, B-cell and monocytic populations [7,9]. Secondly, approximately 80-90% of the NKp46^+ ^cells expressed CD16 (FcγRIII) - the receptor that mediates antibody-dependent cell-mediated cytotoxicity by NK cells - comparable to levels observed in the equivalent populations in cattle and in human PBMC [34,36]. These data indicate that the CD16^+^/CD14^- ^phenotype used recently by Elhmouzi-Younes et al. [21] to define peripheral ovine NK cells successfully identifies most but not all NKp46^+ ^cells and could be equally well applied to NK cells in secondary lymphoid tissues. Thirdly, ovine NKp46^+ ^cells expressed perforin, an integral part of the granule exocytosis mediated cytotoxicity machinery used by NK cells for target cell lysis. The constitutive expression of molecules involved in this cytotoxicity pathway is characteristic of NK cells with lytic function and permits them to function without the requirement for induced expression that is seen in CD8^+ ^T-cells [37,38]. Fourthly, as seen in humans and cattle NK cells, ovine NKp46^+ ^cells exhibited varied levels of CD2 and CD8 expression, such that in blood CD2^-/lo ^and CD2^hi ^as well as CD8^-/lo ^and CD8^hi ^subsets could be distinguished, whilst in secondary lymphoid tissues a more homogenous CD2^lo^/CD8^lo ^population was observed [7,39]. Of note, ovine NKp46^+ ^cells did not express CD11b, a molecule expressed on both human and murine NK cells; a previous report has described bovine NK cells as also having a CD11b^- ^phenotype, suggesting this may be a general characteristic of ruminant NK cells [40].

In this study we have demonstrated that ovine NKp46^+ ^cells isolated from PBMC and cultured in the presence of rOvIL-2/rHuIL-15 exhibited cytotoxicity, one of the "classic" NK cell functions. In direct lysis assays against the xenogenic target cell lines K562 and YAC-1 the levels of cytotoxicity achieved by NKp46^+ ^cells varied between individual sheep, similar to observations made in cattle [7]. However, in redirected lysis assays using the anti-NKp46 antibody, NKp46^+ ^cells from all individuals showed high levels of killing, demonstrating that i) all of the ovine NKp46^+ ^cultures had the capacity to mediate cytotoxicity, with differences of cytotoxicity observed in direct lysis assays therefore likely to represent variation in the repertoire of expressed inhibitory/activatory receptors by NKp46^+ ^cells from individual sheep and ii) as reported in other species engagement of the NKp46 receptor is a potent trigger for NK cell activity [6,7,9]. During culture, NKp46^+ ^cells were non-adherent and became enlarged with prominent lamellopodia (data not shown). These morphological characteristics and the predominantly CD2^lo^/CD8^hi^/CD25^+ ^phenotype, resembled those described for ovine CD16^+^/CD14^- ^cells and bovine NKp46^+ ^cells [21] and provide further evidence that ovine NKp46^+ ^and CD16^+^/CD14^- ^cells represent overlapping populations. Interestingly we noted that cultured NKp46^+ ^cells were CD16^lo^, suggesting downregulation of CD16 during activation, in contrast to the upregulation seen in cattle [34]. This downregulation may contribute to the low level of cytotoxicity reported by Elhmouzi-Younes et al. [21] in re-directed cytotoxicity assays using the anti-CD16 antibody, although data from similar studies in cattle suggest that NKp46 engagement induces higher cytotoxicity than that of CD16 [34].

NK cells are widely distributed throughout both lymphoid and non-lymphoid tissues [41]. Consistent with this, we identified ovine NKp46^+ ^populations in blood, lymph nodes, spleen and at the mucosal surfaces of the lung, gastro-intestinal tract (GIT) and uterus. Within lymph nodes ovine NKp46^+ ^cell distribution corresponded with that described for NK cells in bovine, murine and human lymph nodes - with scattered cells present in the paracortical and medullary regions but largely absent from the follicles [3,8,34,42]. A principal role of lymph node NK cells is to regulate adaptive and innate immunity through participating in a reciprocal activation dialogue with dendritic cells and via the provision of IFNγ for T_H_1 priming of CD4^+ ^T-cells [3,43,44]. Paracortical NK cells are in close proximity to DC and T-cells and optimally situated for this function. In contrast it is thought that medullary NK cells represent cells exiting the lymph nodes and are therefore of limited functional relevance [43]. The distribution of NK cells at the mucosal surfaces has not been described previously in ruminants. NKp46^+ ^cells were found in the mucosa of all compartments of the ovine GIT examined but were not detected in the rumen. Absence of NKp46^+ ^cells in the rumen probably reflects the distinct physiological role of the ruminant fore-stomachs (rumen, omasum and reticulum) which have a stratified epithelial lining and act as a fermentation system. In the rest of the ovine GIT NKp46^+ ^cells were present at high density adjacent to lymphoid aggregates (such as Peyer's patches) and found at a lower density throughout the lamina propria with occasional cells present in an intra-epithelial position. This distribution is similar to that described in mice and humans [45,46] and reflects a functional role of NK cells in interacting with antigen-presenting cells, which are also found predominantly in the lamina propria. NKp46^+ ^cells were also found in sub-epithelial locations in the non-gravid uterus and in both sub-epithelial locations and within the parenchyma of the lungs. Mucosal surfaces, especially those of the gut and lung, are major portals for pathogen entry. NK cells in the lung have been shown to contribute to protection against a wide range of pulmonary pathogens by production of IFNγ and/or cytotoxic activity [47]. In contrast, recent evidence from human and murine studies suggest that the majority of NK cell in the GIT lack IFNγ and cytotoxic function but contribute to mucosal immunity against pathogens by production of IL-22 [45,46]. Although uterine NK cells are also considered to have an anti-pathogenic function, evidence for this is limited [48], with most uterine NK cell studies focusing on their functional role in regulating placental development during pregnancy [49]. However, uterine NK cell biology is complex and the exact role(s) of uterine NK cells remains controversial [48,49]. Futhermore, the significant differences between the reproductive biology of ruminants (cotyledonary-epitheliochorial placental structure) and rodents/primates (discoid-haemochorial placentae) suggest that functional equivalence of uterine NK cells in these species can not be totally assumed.

In conclusion, we have provided a preliminary characterisation of ovine NKp46^+ ^cells and shown them to have a phenotype, distribution and function similar to those of NK cells in other species. Recently there have been rapid advances in understanding of NK cell biology, including the identification of novel functions [5,45,46] and evidence of "memory-like" NK cell subsets [50,51]. The availability of a monoclonal antibody specific for ovine NK cells will hopefully facilitate further studies both of the comparative biology of these cells in different species and also to investigate their role in immunity to important production diseases of sheep.

## Competing interests

The authors declare that they have no competing interests.

## Authors' contributions

TC - design of study, laboratory work and preparation of manuscript; AK - design of study and editing manuscript; AP - production of recombinant protein; NM - generation of the mono-clonal antibody; JB - fluorescent immunohistochemistry; HL - production the recombinant ovine IL2; WIM - design of study and editing manuscript. All authors read and approved the final manuscript.
